# Linking individual variation in facial musculature to facial behavior in rhesus macaques

**DOI:** 10.1002/ar.25650

**Published:** 2025-03-17

**Authors:** Clare M. Kimock, Charles Ritchie, Jamie Whitehouse, Claire Witham, Claire M. Tierney, Nathan Jeffery, Bridget M. Waller, Anne M. Burrows

**Affiliations:** ^1^ Department of Psychology Nottingham Trent University Nottingham UK; ^2^ Department of Musculoskeletal and Ageing Science, Institute of Life Course & Medical Science University of Liverpool Liverpool UK; ^3^ Centre for Macaques, Medical Research Council, Porton Down Salisbury UK; ^4^ Department of Physical Therapy Duquesne University Pittsburgh Pennsylvania USA

**Keywords:** face, facial expression, facial musculature, rhesus macaque

## Abstract

Facial expression is a key component of primate communication, and primates (including humans) have a complex system of facial musculature underpinning this behavior. Human facial musculature is highly variable across individuals, but to date, whether other primate species exhibit a similar level of inter‐individual variation is unknown. Whether individual‐level variation in facial musculature covaries with significant differences in facial movement within the same individual is also unknown. Here, we use facial dissection data from 31 adult rhesus macaques, the largest sample to date, to quantify inter‐individual variation in facial muscle presence. We used a subsample of eight individuals to measure covariation between facial muscle presence and the presence of external facial movements (action units in the Facial Action Coding System, or FACS). We found, in contrast to humans, limited inter‐individual variation in muscle presence, but the zygomatic region exhibited more gross anatomical variation in muscle presence and morphology than any other region of the macaque face. We also found a good correspondence between facial muscle presence and the presence of the associated action units. Our results indicate that the observed variation in rhesus macaque facial expressivity is not likely driven primarily by variation in facial muscle presence but may instead be due to other factors such as learned behavior and/or physiological differences. These findings provide insight into the anatomical basis of inter‐individual variation in facial behavior in primates and suggest potential differences in variation between humans and other primate species.

## 1 INTRODUCTION

Facial expression, the production and coordination of facial movements, is an important component of multi‐modal communication in humans and nonhuman primates (Higham & Hebets, 2013; Waller et al., 2020, 2022). Humans exhibit individual differences in facial expression production, such that people vary in how and the degree to which they produce facial movements (Hildebrandt et al., 2015; Ilgen et al., 2021; Kashyap et al., 2012; Kavanagh et al., 2024). Similarly, humans exhibit marked variation in the facial musculature underpinning facial movement (Abramo et al., 2016; Boyle et al., 2022; Farahvash et al., 2010; Pessa et al., 1998). The extent to which these two levels of variation are related to each other, however, is largely unknown. Likewise, whether these patterns of anatomical and functional variation are unique to humans or shared with other primates is not well understood.

Most studies focus on averages or the universality of facial expressions, which limits our understanding of the proposed functional correlates, constraints, and evolution of inter‐individual variation. Historically, scientists have measured facial expression behavior in humans using an emotion‐based approach, focusing on six “universal” expressions: happiness, fear, disgust, anger, surprise, and sadness (Ekman & Friesen, 1971). This approach is limited because it assumes that facial behavior reflects underlying emotional states, which may not always be the case (Barrett et al., 2019). To avoid attributing emotion and to measure facial movement more objectively, researchers have adopted an anatomically based approach: the Facial Action Coding System (FACS) (Ekman et al., 2002; Ekman & Friesen, 1978), which is now widely used in the social and cognitive sciences (Rosenberg & Ekman, 2020). FACS is comprised of 33 action units (AUs), which describe changes in the position of external facial landmarks produced by contraction or relaxation of the underlying musculature. The coding system also includes 25 action descriptors (ADs) that describe more general movements or movements that involve non‐mimetic muscles (e.g., head/eye position). FACS was developed based on the assumption that muscle movement corresponds to the movement of external facial features and landmarks (Ekman & Friesen, 1978). This assumption has been investigated using intramuscular stimulation (IM) in humans (Efthimiou et al., 2024; Waller et al., 2006), but as collecting in vivo facial movement data and dissection data from the same individuals is not feasible, it has not been possible to check the direct correspondence between precise muscle form and surface appearance changes. To our knowledge, there is no comprehensive published data on the specific variability of AUs across individuals, but automated tracking systems do not reliably identify all AUs (e.g., iMotions extracts 16 AUs, www.imotions.com) and studies using FACS usually focus on a subset (e.g., a study focussing on individual repertoires of AUs only included 11 [Ilgen et al., 2021]). It is possible that the AUs that are not reliably identified using FACS are also those that vary the most across individuals and are sometimes not present. Nevertheless, individuals vary markedly in how expressive they are generally, producing between 10 and 211 AUs per minute (Rollings et al., 2024). This facial expressivity seems to be a stable trait, in that individuals are consistently more or less expressive across contexts similar to other measures of personality (Kavanagh et al., 2024).

The available dissection data show that human facial muscles exhibit marked inter‐individual variation in muscle presence and morphology. The muscles involved in “basic” expressions vary little among individuals, while muscles not involved in these expressions exhibit more inter‐individual variation (Waller, Cray, & Burrows, 2008). Human facial muscles also vary within and between populations. For example, the zygomaticus minor muscle was present in 36% of one cadaveric sample, and the risorius muscle was present in 6% of that same sample (Pessa et al., 1998), while the zygomaticus minor muscle was present in 60% and the risorius muscle was present in 31% of another cadaveric sample (Farahvash et al., 2010). The malaris muscle was present in 54% of a third cadaveric sample (Park et al., 2011). Choi, Hur, et al. (2014) identified four “types” of zygomaticus minor muscle based on differences in attachment site, and Pessa et al. (1998) identified a bifid zygomaticus major muscle in 34% of their sample. Although this variation has been documented, we still lack data on relationships between muscle variation and external facial movement in the same individual, making it difficult to fully validate the “FACS” approach to measuring facial expression.

Comparisons with non‐human primates can help illuminate which aspects of facial anatomy and behavior are predominantly human traits, and which are shared with our closest living relatives. Rhesus macaques (*Macaca mulatta*) are a useful species for comparison because their ecology, behavior, physiology, and morphology have been extensively studied, and they are commonly used in biomedical research because of their anatomical and physiological similarities to humans (Cooper et al., 2022). Like humans, rhesus macaques produce facial movements (expressions) as part of their multimodal communication system (Partan, 2002; van Hooff, 1967). Rhesus macaque facial behavior has also historically been described using stereotyped, prototypical expressions, such as the open mouth threat (van Hooff, 1967), bared teeth/fear grimace (Preuschoft, 2000), and lip‐smack (Ghazanfar et al., 2005; van Hooff, 1967). More recently, MaqFACS (FACS adapted for rhesus macaques) has been used to measure rhesus macaque facial behavior in a more precise way (Rincon et al., 2023), and to make comparisons with human facial behavior (Parr et al., 2010; Waller et al., 2020). MaqFACS is an anatomically based facial movement coding system developed using data from facial dissections (Burrows et al., 2009), intramuscular stimulation (Waller, Parr, et al., 2008), and video footage of facial behavior (Parr et al., 2010). MaqFACS includes 17 AUs, including three ear action units (EAUs) and one AD (lip smacking) (Parr et al., 2010). This system can capture subtle differences in facial behavior that coding of stereotyped expressions cannot (Clark et al., 2020). MaqFACS data have also revealed evidence of individual differences in facial behavior and links between facial behavior and social network position in captive rhesus macaques (Whitehouse et al., 2024). In single male groups, dominant males with greater facial expressivity are better at managing their groups. For example, males with higher AU diversity (the number of different AUs observed) were more centrally positioned in their networks, and those with a higher duration of facial movement overall had more socially cohesive groups. Individual AUs, including AU27 (jaw drop), AU10 (upper lip raiser), and AU16 (lip depressor) were also predictive of centrality (Whitehouse et al., 2024).

Rhesus macaques exhibit a similar number and configuration of facial muscles to other catarrhine primates, including humans (Burrows et al., 2009; Diogo et al., 2009; Huber, 1933) and MaqFACS has been used successfully to measure facial behavior in rhesus macaques and other macaque species (e.g., Barbary macaques [*Macaca sylvanus*] [Julle‐Danière et al., 2015], Japanese macaques [*Macaca fuscata*] [Correia‐Caeiro et al., 2021], and crested macaques [*Macaca nigra*] [Clark et al., 2020]). However, we still do not know whether there is marked variation in the configuration of facial muscles between individuals, and how this might relate to facial behavior across individuals. Data on the latter are very difficult to collect because both antemortem behavioral observations and post‐mortem dissection data from the same individuals are required. Some authors describe “distinct” rhesus macaque facial muscles (Burrows et al., 2009), while others report some blending of muscle fibers or “sheet‐like” muscle morphology (Huber, 1933). For example, Burrows et al. (2009) described the zygomaticus major muscle, the variably present zygomaticus minor muscle, and the orbicularis oculi muscle as separate muscles, while Huber (1933) described a “zygomatico‐orbito/zygomaticus muscle mass.” Yet prior studies have been based on small samples sizes (e.g., sample size unspecified [Huber, 1933], six individuals [Burrows et al., 2009], 10 individuals, some of which were published previously [Diogo et al., 2009]). Larger samples are required to understand the extent of inter‐individual variation in rhesus macaque facial muscle presence and morphology, and the ways in which these individual differences in anatomy might impact facial behavior, and in turn, social behavior and relationships. Facial expression is highly developed in primates, and particularly humans, and key to understanding the evolution of this specialized trait is understanding how it exists alongside complex variability of underlying anatomical structures.

Here, we aimed to understand how individual differences in facial musculature might influence facial behavior in rhesus macaques. We integrated anatomical and behavioral data from a captive population of rhesus macaques to (1) describe individual differences in facial musculature in terms of muscle presence and morphology and (2) investigate intra‐individual relationships between facial musculature and facial behavior by measuring the correspondence between the presence of AUs and the presence of their proposed muscular basis within the same individual.

## 2 MATERIALS AND METHODS

### 2.1 Study site and subjects

We conducted this study using rhesus macaque specimens from the Medical Research Council Centre for Macaques (CFM). The CFM is a captive breeding facility in Porton Down, United Kingdom (UK), which is managed by the UK Home Office. The CFM rhesus macaque population comprises about 200 individuals, housed in single male breeding groups (one male, two to eight females) and single‐sex non‐breeding stock groups. The CFM houses these groups in large enclosures (a main enclosure measuring 8.0 m × 3.4 m × 2.8 m and an adjoining caging area measuring 6.5 m × 1.5 m × 2.8 m), provides food (complete primate diet, fruit and vegetables and forage mix) once a day, water ad libitum, and enrichment. All individuals in the present sample were culled for purposes unrelated to the aims of the current project (such as poor health and/or population management) by administration of an overdose of anesthetic agent (pentabarbital sodium). We requested post‐mortem tissue from deceased animals between 2020 and 2024. Our sample consisted of 31 adult individuals, including 26 females (ages 4.24 to 15.40 years) and 5 males (ages 4.71 to 19.53 years).

### 2.2 Ethics statement

Video data collection was conducted under protocols approved by the Animal Welfare Research and Ethics Board of the Centre for Macaques (ethics number CFM2020E001, Home Office site license PEL number: X809B70BC) and the Animal Welfare Research and Ethics Board at Nottingham Trent University. Post‐mortem tissue dissection was approved by the University of Liverpool Animal Welfare Research and Ethics Board (ethics number AWC0245) and the Animal Welfare Research and Ethics Board at Nottingham Trent University.

### 2.3 Facial musculature

#### 2.3.1 Dissection procedures

Following euthanasia, CFM staff conducted necropsies during which they disarticulated the head from the cervical part of the vertebral column, removed the brain and eyes, and stored the disarticulated heads in buckets filled with 10% buffered formalin. Specimens were shipped to the Human Anatomy Resource Centre (HARC) at the University of Liverpool and subsequently placed in a solution of 50:50 methanol and water, in keeping with standard practices at HARC.

We used a posterior‐anterior (P‐A) or “reverse dissection” method (c.f. Burrows et al., 2009, 2019) to remove the facial mask from the skull and to visualize the facial musculature. We used disposable #11 scalpels, periosteal elevators, and forceps to detach the facial mask following the edges of tissue incised for removal of the brain (e.g., superior to the orbits/glabella and posterior to the ears) and disarticulation of the head during necropsy (e.g., inferior to the mandible). Starting at the superior incision, we lifted the periosteum from the bone using periosteal elevators until we reached the temporal fascia. We then removed the tissue superficial to the temporal fascia until we reached the zygomatic arch. We scraped the periosteum off the zygomatic arch, working inferiorly and medially until we reached the midline (nasal region). We also worked medially from incisions caudal to the ear, following the fascial layer over the parotid gland and masseter muscle, leaving the parotid gland and masseter muscle behind, attached to the skull. From the incisions inferior to the mandible, we dissected in a superior direction, detaching the platysma muscle up to the inferior aspect of the mandible. We removed the oral mucosa along with the mask. We worked medially from all incision points until we could detach the facial mask from the bone at the orbital and nasal margins. We removed the entire mask in one piece instead of two sides separated at the midline as in Burrows et al. (2009, 2019) because we wanted to assess intra‐individual variation, including bilateral asymmetry.

There were some inconsistencies in muscle preservation, primarily in the platysma and frontalis muscles, due to variation in necropsy methods. After the masks were detached from the bone and deep fascial layers, we pinned the mask edges to a silicone mat and used scalpels to remove the fascia, connective tissue, nerves, and vasculature, revealing the facial musculature. We reflected some of the deeper musculature (e.g., buccinator and levator anguli oris muscles) to reveal the more superficial insertions of the zygomaticus muscles.

**FIGURE 1:**
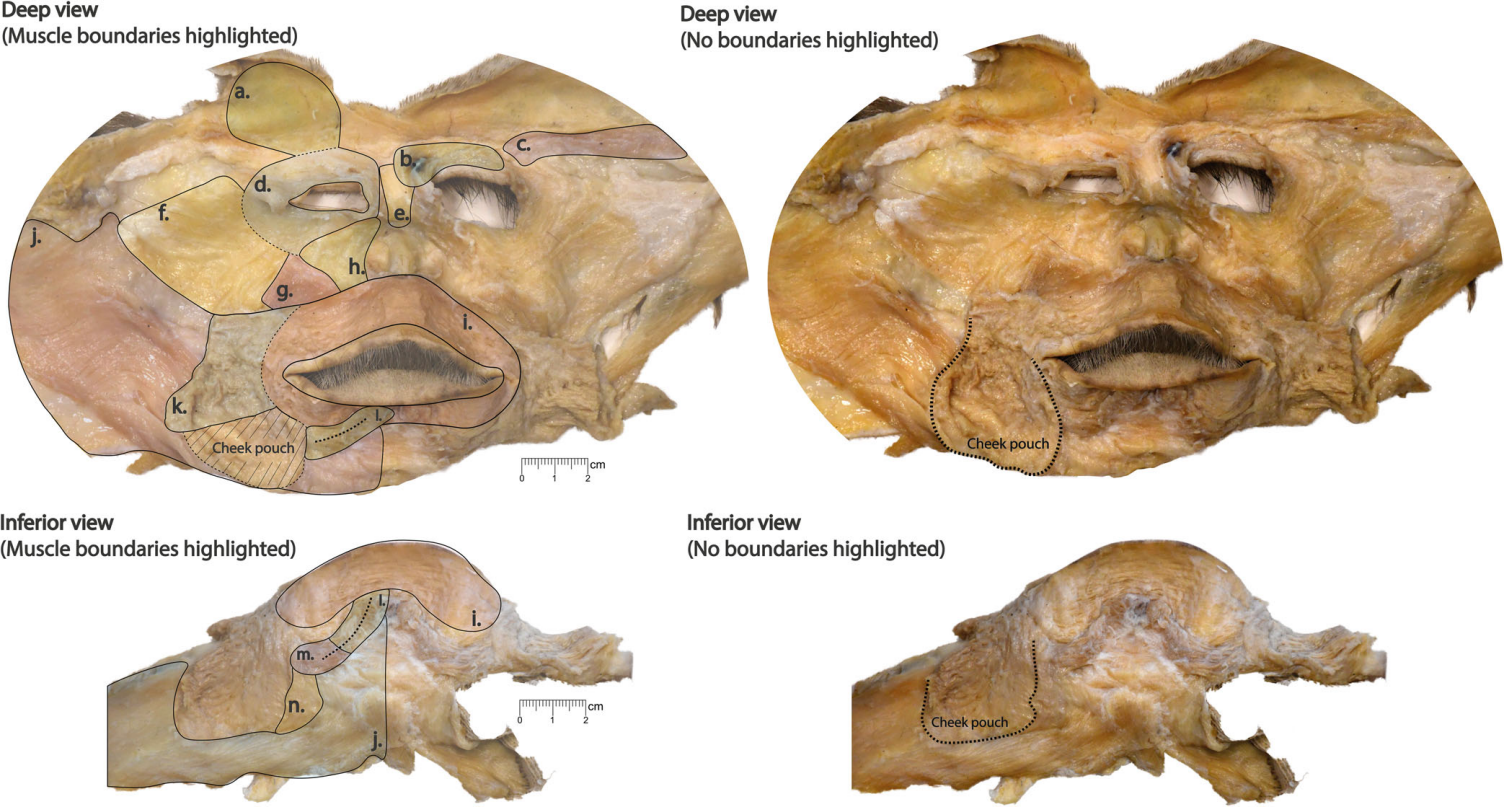
Image of deep and inferior‐deep views of a facial mask, labeled with muscles of facial expression examined in this study. (a) Frontalis, (b) corrugator supercilii, (c) orbitoauricularis, (d) orbicularis oculi, (e) procerus, (f) zygomatic region, (g) levator anguli oris, (h) levator labii superioris + levator labii superioris alaeque nasi, (i) orbicularis oris, (j) platysma, (k) buccinator, (l) mentalis, (m) depressor anguli oris, (n) depressor labii inferioris.

We collected images of the cleaned facial masks using a Nikon D7500 with the AF‐S DX Nikkor 18–140 mm f/3.5–5.6G ED VR lens. We mounted the camera on a tripod, taking care to keep the lens as parallel to the specimen as possible. We pinned the specimens to a silicone mat on a white tray, propping up the specimen with muslin cloth where necessary. We included a metric scale in each image. We enhanced image contrast using the auto contrast function in ImageJ (Schneider et al., 2012) so that muscle fibers were easier to see. An image of one cleaned facial mask is presented in Figure 1.

#### 2.3.2 Measuring individual differences in facial musculature

Three observers (CMK, CR, and AMB) collected data on muscle presence directly from the adult specimens with the aid of a lighted 5× magnifying lens mounted to the edge of the dissecting table. The muscles examined in this study are presented in Figure 1. We scored bilateral muscles on both sides (left and right) for a total of 62 sides across the 31 specimens. Observers considered fiber location, orientation, and attachment when assessing muscle presence. We removed the bony origins as part of the dissection process, but we did use soft tissue insertions (where visible) to determine presence. We used published descriptions from Huber (1933) and Burrows et al. (2009), which we present in Table 1, as a guide. Muscle descriptions are based on a combination of anterior–posterior and posterior–anterior dissections (Burrows et al., 2009; Burrows, Waller, & Micheletta, 2016; Huber, 1933). Since we only used the posterior–anterior method, we could not always assess all previously described origins and insertions. We did not score the depressor supercilii or nasalis muscles due to difficulties in visualizing them using the mask method. We also excluded the incisivii muscles, which are the proposed muscular basis of AU18i and AU18ii (the lip pucker AUs) (Parr et al., 2010) as Huber (1933) noted that these muscles were not distinct in the rhesus macaque, and we also could not clearly define their presence or morphology. Presence scores (present/not present) were based on a consensus among the three observers. To assess inter‐ and intra‐individual differences in facial musculature, we compared muscle presence across specimens and within specimens.

**TABLE 1:** Facial muscles scored for presence in this study.

Muscle	Origin	Insertion	Typical appearance
Frontalis	Galea aponeurotica	Fascia in superciliary region, and corrugator supercilii and orbicularis oculi muscles	Gracile fibers running inferiorly over the frontal bone to the superior margin of the orbits
Procerus^‡^	Nasal bones, superficial dermis in the nasal region	Frontalis muscle	Gracile fibers running supero‐inferiorly from the glabellar region to the nasal region
Corrugator supercilii	Bony origin in the medial palpebral region, lateral to glabella	Dermis of superciliary region including inferior border of frontalis and fibers of orbicularis oculi	Robust, rope‐like fibers in a fan‐shape running along superior margin of the orbits
Orbicularis oculi	Medial palpebral region	Orbitoauricularis, corrugator supercilii^†^, and zygomatic minor muscles	Gracile fibers in a sphincter shape surrounding the orbit and palpebral regions
Orbitoauricularis	Superolateral orbital region	Dermis near superorostral pinna, orbicularis oculi, frontalis, and auricular muscles, galea aponeurotica	Robust, “rope‐like” fibers oriented rostro‐posteriorly between the orbit and the auricle
Zygomaticus major	Zygomatic arch and fascia superficial to the zygomatic arch	Orbicularis oris at the modiolus^a^, with fibers splitting around the levator angli oris and depressor anguli oris muscles (the lateral fibers insert deep to orbicularis oris)	Sheet‐like fibers running inferio‐medially across the midface
Zygomaticus minor	Dermis superficial to inferior rim of orbit	Fibers of depressor anguli oris muscle	Sheet‐like fibers running inferio‐medially, between the zygomatic major and orbicularis oculi muscles
Zygomatico‐orbital muscle mass^†^	Zygomatic arch and margin of orbit	Modiolar region^a^, where buccinator, levator anguli oris, and depressor anguli oris muscles intersect	Sheet‐like fibers running inferio‐medially with no clearly defined bands
Levator labii superioris	Nasal and maxillary bones and medial palpebral region	Dermis superficial to nasal and maxillary bones and upper fibers of orbicularis oris muscle	Fibers running inferiorly between the orbit and lip, lateral to the nose
Levator labii superioris alaeque nasi	Nasal and maxillary bones and medial palpebral region	Dermis on lateral border of the nasal ala	Fibers running inferiorly between orbit and upper lip medial to the levator labii superioris muscle
Levator anguli oris (caninus)	Canine fossa of the maxilla	Modiolus^a^ (orbicularis oris muscle)	Robust, oblique fibers superior to the orbicularis oris muscle, which form a triangular shape
Buccinator^†^	Alveolar margin of maxilla and mandible, pterygomandibular ligament	Modiolus^a^	Robust fibers running rostrocaudally from the corner of the mouth
Orbicularis oris	Zygomatic muscles (major/minor/mass), levator labii superioris muscle, and alveolar margin of the maxilla	Platysma, depressor anguli oris, depressor labii inferioris, buccinator, and mentalis muscles, and alveolar margin of the mandible	Multiple layers of robust sphincter fibers surrounding the oral region
Platysma	Nuchal crest and skin over the lateral aspect of the face and neck	Levator anguli oris muscle, fibers of the orbicularis oris, depressor anguli oris, depressor labii inferioris and mentalis muscles	Sheet‐like thin fibers extending over the face and neck
Mentalis	Lower fibers of orbicularis oris muscle, alveolar region of the mandible^†^	Superficial dermis in mental region	Robust fibers running rostrocaudally
Depressor anguli oris (triangularis)	Fascia superficial to orbicularis oris and inferior border of the zygomaticus muscle(s)	Modiolus^a^, inferior fibers of orbicularis oris, platysma, and levator anguli oris^†^ muscles	Gracile fibers curving around corner of the mouth
Depressor labii inferioris	Lower fibers of orbicularis oris muscle	Dermis superficial to the mental region	Gracile fibers

*Note*: Definitions are based on data presented in Burrows et al. (2009), Burrows, Rogers‐Vizena, et al. (2016) and where denoted with a “†,” Huber (1933), or “‡,” modified from descriptions in humans (Boyle et al., 2022).

^a^ Modiolus (and modiolar region) refers to the fibrous convergence of muscular insertions at the corner of the mouth.

### 2.4 Intra‐individual relationships between musculature and behavior

#### 2.4.1 Facial behavior

Two observers collected ad lib antemortem video footage of facial behavior as part of a separate project on individual differences in facial behavior. Sixteen individuals in the dissection sample were filmed as part of this separate project. We positioned a Panasonic HDC‐SD700 (filmed at 50 fps, resolution: 1920 × 1080) camera outside of the glass enclosure and recorded video footage when individuals were visible. To maximize the ability to detect detail in subsequent coding, videos were zoomed in on the face of the animal during filming, and videos were encoded with high‐definition codecs during processing. As videos were opportunistic, they included a range of different non‐social and social contexts, including aggression, affiliation, close physical proximity to others, grooming, foraging, and vigilance. Most contexts were seen across all individuals, except for affiliation, which was rare (two individuals only). Video clips ranged from 30 s to 10 min in length.

Two separate observers, who were not involved in the video data collection, conducted frame‐by‐frame MaqFACS coding of video footage using BORIS video coding software (Friard & Gamba, 2016). Our coding protocol included all action units described in Parr et al. (2010), and we added AD 50 for chewing (in which lower‐face masticatory movements were ignored, but coding continued for the rest of the face) (Whitehouse et al., 2024). AUs, their definitions, and their proposed muscular basis are presented in Table 2. In this study, we excluded AU26 and AU27 because these actions are produced by a combination of facial and non‐facial muscles (the digastric muscles). Using the facial mask technique, we could not visualize the digastric muscles to investigate the entire muscular basis of AU26 and AU27. We also excluded AU18i and AU18ii due to difficulties in defining and identifying the incisivii muscles, as described above. Observers coded frames where an individual's face was sufficiently visible to code facial behavior (e.g., at least half of the face was in view of the camera, and where all AUs could be reliably observed). We used the total number of codable frames to calculate observation time (in seconds). Observers coded the first and last frames during which an animal produced an AU. We extracted AU frequency (the number of times an action unit was produced) from the coded frames. Frequencies for each AU were then converted to rates (i.e., action unit per minute) by dividing them by the total observation time. Both of our coders were certified MaqFACS coders (certified externally by achieving at least 0.7 Wexler's agreement on a standardized coding task). In addition, we calculated an interclass correlation to assess for interrater reliability of AU frequency specific to our data by using 10 randomly selected videos (approximately 3% of the dataset, function ICC in R package psych), each of which were coded independently. We found moderate agreement between our coders (average ICC: 0.67, *p* ≤ 0.001). Within FACS, an agreement of above 0.6 is considered appropriate, with agreement values approaching 0.7 being considered excellent. For our analyses, we converted rates into presence (0/1, whether the AU was observed at least once).

**TABLE 2:** Table of AUs examined in this study and their definitions/proposed muscular basis (definitions from Parr et al., 2010; Waller et al., 2020).

Action unit	Definition	Proposed muscular basis
AU1 + 2	Brow raiser	Frontalis
AU41	Glabella lowerer	Procerus
AU6	Cheek raiser	Orbicularis oculi (pars orbitalis)
AU8	Lips toward each other	Orbicularis oris
AU9 + 10	Nose wrinkle + upper lip raiser	Levator labii superioris alaeque nasi + levator labii superioris
AU10	Upper lip raiser	Levator labii superioris
AU12	Lip corner puller	Zygomatic major
AU16	Lower lip depressor	Depressor labii inferioris
AU17	Chin raiser	Mentalis
AU25	Lips parted	Orbicularis oris + depressor labii inferioris + levator labii superioris

We also conducted two exploratory analyses: one to determine the minimum observation time required to observe each action unit (see Supporting Information for methodology), and another on asymmetry in facial behavior. Most AUs were observed after an hour, so we set an hour as the minimum threshold for inclusion in the behavior and anatomy correspondence analysis (Figure S1). Of the 16 individuals in the dissection sample that were filmed, eight individuals met the minimum threshold for inclusion. For the asymmetry analysis, a third observer re‐coded a subset of videos (~10% of the available footage for the subset of eight animals which met the threshold for inclusion in the behavioral analyses) for asymmetry in AU expression. Where an AU associated with a paired muscle had been identified in the primary coding, the third coder scored that AU as: intensity greater on the left side, intensity greater on the right side, or equal intensity on both sides (no asymmetry). We extracted AU duration (the number of seconds for which the AU was produced) on each side (left and right) from the re‐coded data. We chose to use AU duration because it could capture the degree of asymmetry in the AUs better than AU frequency.

#### 2.4.2 Comparisons between musculature and behavior

We made qualitative comparisons between muscle presence and AU presence for the eight individuals (one male, seven females) for whom sufficient behavioral data were available, following the framework laid out in MaqFACS (Table 2). For each individual, we noted all muscles and AUs present and scored (1) whether the corresponding muscle and AU were both present, (2) whether the muscle was present, but the corresponding AU was not, (3) whether the muscle was not present, but the corresponding AU was present, (4) whether muscle presence was unknown but the corresponding AU was present, or (5) whether neither the muscle nor the corresponding AU was present.

## 3 RESULTS

### 3.1 Individual differences in facial musculature

There were few intra‐ and inter‐individual differences in facial muscle presence among the individuals in our sample. The orbicularis oculi, orbitoauricularis, levator labii superioris, levator labii superioris alaeque nasi, levator anguli oris, buccinator, platysma, mentalis, depressor anguli oris, and depressor labii inferioris muscles were present bilaterally in all (31/31) individuals, and the orbicularis oris muscle was also present in all (31/31) individuals (Table 3). The corrugator supercilii muscle was present bilaterally in all (31/31) specimens, but we noted that its fibers tended to be in a vertical orientation. The frontalis muscle was present in most specimens (29/31), but we could not reliably identify it in two of the specimens in which the superior region of the face had been destroyed during necropsy (Table 3). We also identified the procerus muscle in most, but not all (29/31), of the individuals (Table 3). It is easier to visualize the deeper musculature using the posterior–anterior (reverse dissection) technique, but we could only reveal small portions of the more superficial muscles, such as procerus and depressor anguli oris.

**TABLE 3:** Facial muscle presence data, excluding the zygomatic region (*n* = 31 individuals, 62 sides).

Muscle	Presence (left)	Presence (right)
Frontalis^a^	29/31 (94%)	29/31 (94%)
Corrugator supercilii	31/31 (100%)	31/31 (100%)
Orbicularis oculi	31/31 (100%)	31/31 (100%)
Orbitoauricularis	31/31 (100%)	31/31 (100%)
Levator labii superioris	31/31 (100%)	31/31 (100%)
Levator labii superioris alaeque nasi	31/31 (100%)	31/31 (100%)
Levator anguli oris	31/31 (100%)	31/31 (100%)
Buccinator	31/31 (100%)	31/31 (100%)
Platysma	31/31 (100%)	31/31 (100%)
Mentalis	31/31 (100%)	31/31 (100%)
Depressor anguli oris	31/31 (100%)	31/31 (100%)
Depressor labii inferioris	31/31 (100%)	31/31 (100%)

Muscle	Presence (midline)
Procerus^a^	29/31 (94%)
Orbicularis oris	31/31 (100%)

^a^ Presence unknown for specimens not scored as “present.”

Muscle fibers often blended with the surrounding musculature, making it more challenging to delineate muscle boundaries. The levator labii superioris alaeque nasi muscle was difficult to distinguish from the levator labii superioris muscle without the use of a magnifier and in photographs of the specimens. Fibers of the depressor anguli oris muscle blended with fibers of the mentalis muscle, and the depressor labii inferioris muscle blended with the platysma muscle such that it often appeared to be an extension of the platysma muscle.

**FIGURE 2:**
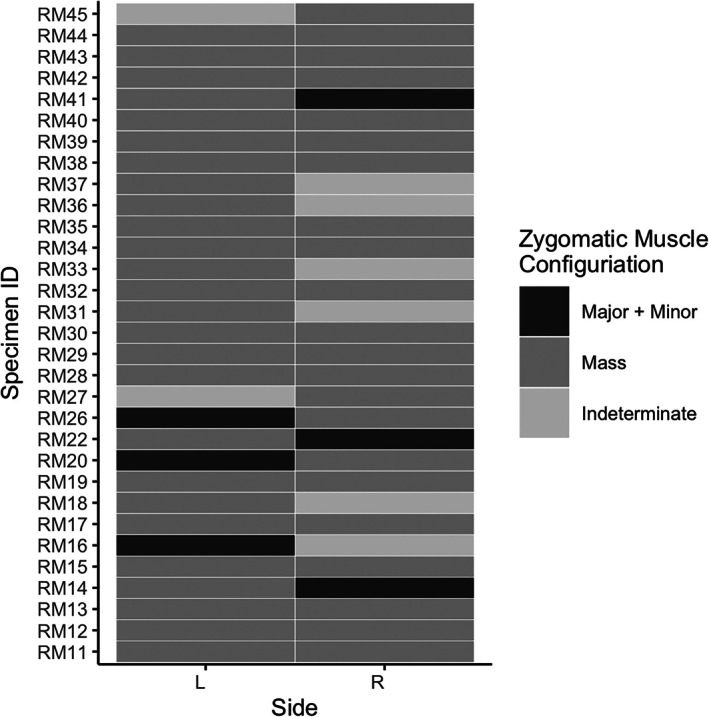
Heatmap of muscle presence data for the zygomatic region (*n* = 31).

**FIGURE 3:**
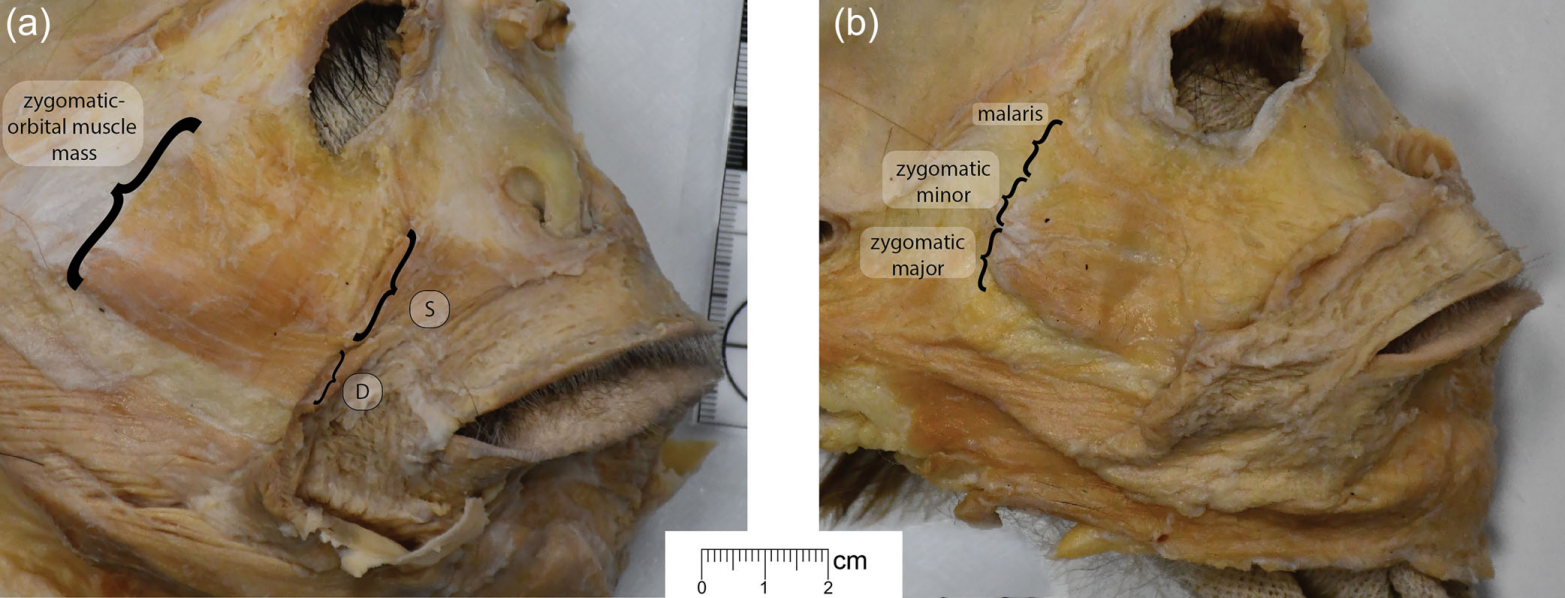
Examples of gross‐level anatomical variation in the zygomatic region. (a) Deep view of the facial mask of an individual with a zygomatico‐orbital muscle mass. (b) Deep view of the facial mask of an individual with zygomaticus major and zygomaticus minor muscles, and a possible malaris muscle. S = superficial, D = deep.

The zygomatic region exhibited gross‐level inter‐individual variation in muscle presence and configuration (Figures 2 and 3). In most specimens (48/62 sides, or 77% of sides), there were continuous muscle fibers from the zygomaticus major muscle to the orbicularis oculi muscle, forming a sheet or “mass,” as described by Huber (1933). A fully separate zygomaticus major muscle was present only in 7 out of 62 sides, and a separate zygomaticus minor muscle was present in only 6 out of 62 sides. In 8 out of 62 sides, we could not determine whether the muscle fibers were continuous from the zygomatic region to the orbicularis oculi muscle or not. We did not include the malaris muscle, an accessory band of the orbicularis oculi muscle found in some human specimens (Boyle et al., 2022), in our scoring protocol because it had not previously been documented in rhesus macaques, but we did identify two specimens where this muscle might have been present. We also noted one bilateral occurrence of a bifid zygomaticus major muscle insertion, in which fibers of the zygomaticus major muscle extended laterally to the levator anguli oris muscle. Zygomatic region morphology was generally symmetrical, but we did observe some bilateral asymmetry (Figure 2). Although we observed anatomical variation in this region, we grouped all variants of the zygomatic muscle (major, minor, mass) when we considered correspondence between muscle presence and AU presence below.

We do not present any statistical analyses here because presence for all muscles except for frontalis, procerus, and the zygomaticus muscles was 100% (no inter‐individual variation). Scoring for the frontalis and procerus muscles was also influenced by preservation and potentially by dissection method, so our confidence in scores where we could not identify the muscle is low. We therefore have presented our results as presence/non‐presence.

### 3.2 Intra‐individual relationships between musculature and behavior

#### 3.2.1 Facial behavior

Our first exploratory analysis indicated that most AUs were observed after an hour; we therefore decided to only include individuals with at least an hour of codable video footage (*n* = 8) in the behavioral analyses (Supporting Information). Additionally, neither of the individuals with a possible malaris muscle, nor the individual with the bifid zygomaticus major insertion, was included in the behavioral sample. The eight individuals with associated MaqFACS data exhibited some individual differences in action unit presence (Table 4). All individuals produced AU1 + 2 (brow raiser), AU41 (brow lowerer), and AU25 (lips parted). Most individuals produced AU9 + 10 (nose wrinkler + upper lip raiser), AU10 (upper lip raiser), AU12 (lip corner puller), AU6 (cheek raiser) and AU16 (lower lip depressor). Fewer individuals were observed with AU8 (lips toward each other). Only two individuals generated AU17 (chin raiser). The individual with the lowest observation time (61 min) produced the fewest AUs.

**TABLE 4:** AU presence in the subsample of individuals with associated MaqFACS data (*n* = 8).

Specimen	AU1 + 2	AU41	AU6	AU8	AU9 + 10	AU10	AU12	AU16	AU17	AU25	Expressivity score	Observation time (minutes)
RM29	1	1	1	0	0	0	0	0	0	1	5.507	61.199
RM30	1	1	1	1	1	1	1	1	0	1	13.864	70.900
RM31	1	1	1	1	1	1	1	1	0	1	9.833	91.016
RM32	1	1	1	1	1	1	1	1	1	1	17.699	75.089
RM33	1	1	0	1	1	1	1	1	1	1	5.720	95.629
RM37	1	1	1	1	1	1	1	1	0	1	9.226	81.399
RM38	1	1	1	1	1	1	1	1	0	1	7.961	83.909
RM40	1	1	1	0	1	1	1	1	0	1	9.568	65.214

*Note*: “1” denotes that the AU was observed at least once, and “0” indicates that the AU was not observed. Expressivity score refers to the rate of AUs produced per minute (the frequency of 10 AUs, divided by observation time). Observation time refers to the total minutes of codable footage (the individual's face was sufficiently in view to code AUs).

Our second exploratory analysis on asymmetry in AU expression revealed that few AUs were expressed asymmetrically. Only AU1 + 2 (brow raiser), AU10 (upper lip raiser), AU12 (lip corner puller), and AU16 (lower lip depressor) exhibited any asymmetry. This asymmetry was minor, occurring in a small percentage of the total time the AU was expressed (Figure S2). The greatest asymmetry was in AU12. We therefore decided not to score left and right AUs separately. For our analyses on relationships between anatomy and behavior, we decided to consider a muscle present if it was present on at least one side to be consistent with the AU scoring.

#### 3.2.2 Correspondence between muscle presence and AU presence

**FIGURE 4:**
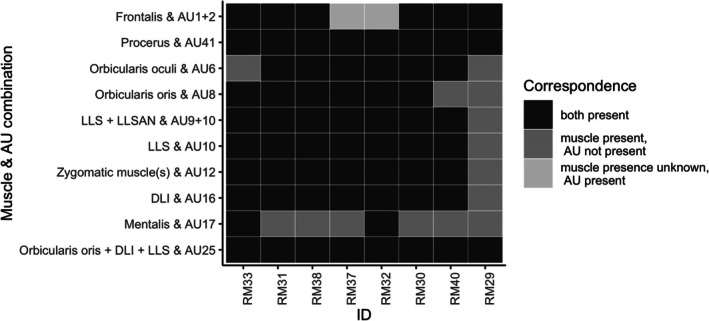
Correspondence between action unit presence and presence of its proposed muscular basis for AUs examined in this study (*n* = 8). Muscle abbreviations: DLI, depressor labii inferioris muscle; LLS, levator labii superioris muscle; LLSAN, levator labii superioris alaeque nasi muscle; Zygomaticus muscle(s), zygomaticus major muscle, zygomaticus minor muscle, and/or zygomatico‐orbital muscle mass. Frontalis was insufficiently preserved to code presence in RM32 and RM37. Individuals are listed in order of observation time (greatest on the left [96 min], least on the right [61 min]).

In most cases where an AU was observed, the corresponding muscle was also present. In 64/80 (80%) of muscle‐AU pairs, both the AU and the corresponding muscle were present (Figure 4). In 14/80 (17.5%) of muscle‐AU pairs, the AU was not recorded, but the corresponding muscle was present. There were two instances (2.5% of muscle‐AU pairs) where an AU was observed, but muscle presence was unknown due to specimen preservation. In both cases, the AU was AU1 + 2 (brow raiser), and frontalis was insufficiently preserved to score its presence. AU17 (chin raiser) was only observed in two individuals, but mentalis, the corresponding muscle, was present in all individuals. Our supplemental analysis also showed that AU17 was the only AU with a relatively low detection rate at 1 h of observation time. In the individual with the lowest observation time, all muscles were present, but only three AUs of interest were observed. We do not present statistical analyses here due to our small sample size (*n* = 8 individuals).

## 4 DISCUSSION

There was little inter‐individual variation in facial muscle presence in our sample of 31 adult rhesus macaque individuals, the largest sample of nonhuman primate facial dissections from a single species to date. Most facial muscles were present in most individuals. However, the midface, especially the zygomatic region, exhibited more gross anatomical variation than any other region of the face. Our sample of macaques exhibited less inter‐individual variation in muscle presence than has been reported in humans (Waller, Parr, et al., 2008). In the subsample of eight individuals for which behavioral data were available, there was good correspondence between AU presence and the presence of the AU's proposed muscular basis. If an action unit was present, its corresponding muscle was present in all cases where sufficient tissue was preserved to score the muscle. Rhesus macaques exhibit inter‐individual variation in facial “expressivity” (the configurations of AUs produced) (Whitehouse et al., 2024), but our data suggest that this observed variation in facial behavior might not be the result of individual differences in facial muscle presence. Instead, these individual differences might be due to more subtle differences in muscle morphology than we can document here, or to physiology, behavior, or social environment. Our results provide insight into the anatomical basis of facial behavior in rhesus macaques and, by extension, the evolution of facial communication in primates.

There was limited inter‐individual variation in muscle presence in our sample. Nearly all muscles examined in this study were present in all individuals. The only muscles that varied in presence were the procerus and frontalis muscles. However, poor preservation due to necropsy procedures (tissue superior to the orbits was destroyed during brain removal) was likely a factor in presence scoring for the frontalis muscle. The procerus muscle is gracile and located superficial to the corrugator supercilii muscle, which may have impacted our ability to visualize it using the mask technique. Our muscle presence results contrast slightly with those from previous studies on rhesus macaque facial musculature (Burrows et al., 2009; Huber, 1933). We found that the orbitoauricularis muscle was present bilaterally in all 31 individuals in our sample, while Burrows et al. (2009) reported that this muscle was variably present (present in 3/5 individuals). We also identified the levator labii superioris alaeque nasi muscle bilaterally in all 31 individuals, but it was variably present (2/5 individuals) in the Burrows et al. (2009) sample. Huber (1933) described a “nasiolabialis” muscle, a sheet‐like muscle extending over the nasal region inferiorly to the lip. We noted that fibers of the levator labii superioris alaeque nasi muscle often blended with the levator labii superioris muscle, making the muscles difficult to distinguish without the aid of a magnifier. The depressor anguli oris muscle also appeared to blend with fibers of the mentalis muscle.

We identified gross anatomical variation in muscle morphology in the zygomatic region, including some bilateral asymmetry. We observed a clear zygomaticus major muscle on eight sides, and a clear zygomaticus minor muscle (Burrows et al., 2009) on six sides. Most sides (48/62) exhibited continuous fibers from the region of the zygomaticus major to orbicularis oculi muscles, morphology more consistent with the “zygomatico‐orbito muscle mass” defined by Huber (1933). This gross anatomical variation was generally symmetrical, but we identified clear bilateral asymmetry in five individuals, such that there were zygomaticus major and zygomaticus minor muscles on one side and a zygomatico‐orbital muscle mass on the other. In a few specimens, we could not confidently assign a description to the morphology in the zygomatic region because it was unclear whether separation in muscle fibers was anatomical variation or a dissection artifact. We also identified two possible instances of the malaris muscle and one bifid zygomaticus major muscle.

Limited data are available on facial musculature in other *Macaca* species. Most comparative studies report data from a small sample of individuals (Diogo et al., 2009). To our knowledge, there is one other detailed study of facial musculature in a *Macaca* species, the crested macaque (Burrows, Waller, & Micheletta, 2016). Results from this study on dissections of three crested macaques indicated that auricular muscles and the levator anguli oris [caninus] muscles were more robust in rhesus macaques than in crested macaques, but the levator labii superioris muscle was broader in crested macaques than in rhesus macaques (Burrows, Waller, & Micheletta, 2016). None of the crested macaques examined exhibited a zygomaticus minor muscle (Burrows, Waller, & Micheletta, 2016). Although we consistently identified fibers in the zygomatic region in our rhesus macaque sample, it was challenging to delineate boundaries. Future studies on larger samples of other *Macaca* species will reveal whether the limited inter‐individual variation we observed in our sample is observed in other closely related species.

In our sample, there was good correspondence between AU presence and the muscle(s) proposed to generate that AU. The zygomatic region exhibited the greatest asymmetry in both muscle morphology and action unit expression, although asymmetry was minimal. The zygomaticus muscles draw the corner of the mouth upwards (AU12, lip corner puller, in MaqFACS). In macaques, this movement forms part of the “bared‐teeth display” (Clark et al., 2020; Preuschoft, 2000). Bared‐teeth displays exhibit some variation in overall form within and between individuals in crested macaques (Clark et al., 2020), but whether subtle differences in the appearance of AU12 might impact the appearance of the display is unknown. The morphological variation we observed in the zygomatic region might impact the form of AU12, and by extension, bared‐teeth displays.

There were two cases where an AU was coded as present, but the presence of the corresponding muscle was unknown. In both cases, the muscle (frontalis) was insufficiently preserved to score its presence. All individuals had the proposed muscular basis for AU6 (cheek raiser), AU8 (lips toward each other) and AU17 (chin raiser), but some individuals never produced these AUs. We simply may not have observed these AUs during our behavioral observations; measuring AU presence reliably is challenging and requires a substantial amount of video data. Even with a 1‐h threshold for inclusion in the behavioral comparisons, it is possible that we did not have sufficient data to detect the more rarely used AUs.

We also made some anatomical observations that may have implications for the use of MaqFACS. First, MaqFACS contains an AU41 (glabella lowerer), the muscular basis of which is the procerus. The procerus is a gracile muscle, but all the individuals in our sample had robust corrugator supercilii muscles, which move the brows toward the midline (AU4) in humans. We noted that the corrugator supercilii fibers in our sample of rhesus macaques were more vertically oriented than these fibers are in humans. Therefore, the external appearance changes associated with these muscles might vary between rhesus macaques and humans. There is no action unit associated with the corrugator supercilii muscles in MaqFACS, but it might contribute to the action of AU41 (glabella lowerer). It might be worth exploring the morphology of the glabella/brow lowering action in macaques further to determine whether the corrugator supercilii muscles are involved in this movement, given their robust morphology and more vertically oriented fibers. If our findings are validated, the proposed muscular basis of AU41 could be updated to include the corrugator supercilii muscle. We also noted that the fibers of the levator labii superioris and the levator labii superioris alaque nasi fibers tended to blend. If these muscles also act together, this finding validates the use of the combined AU9 + 10 code in MaqFACS.

The zygomatic region exhibited the greatest gross anatomical variation in our sample. Many individuals had a continuous mass of fibers stretching from the region of the zygomaticus major to the orbicularis oculi muscle. Currently, there is one action unit associated with the zygomaticus major muscle, AU12 (lip corner puller). When the zygomaticus major muscle was stimulated in a single rhesus macaque, there were no movements of the surface of the face surrounding the eye that would correspond to orbicularis oculi, which might be expected if the fibers were continuous (Waller et al., 2008). If AU12 is produced by the “zygomatico‐orbito muscle mass” or the combined action of the zygomaticus minor, and zygomaticus major (if present) muscles, the proposed muscular basis for AU12 could be updated to include zygomaticus minor. We observed two possible occurrences of the malaris muscle in our sample. To our knowledge, the malaris muscle has not previously been documented in macaques. In humans, the malaris muscle is proposed to produce “crow's feet” around the eyes (Park et al., 2011; Kampan et al., 2019). In macaques, these fibers might contribute to AU6. We also observed one bifid zygomaticus major muscle. In humans, a bifid zygomaticus muscle may produce cheek dimples (Pessa et al., 1998). It is also possible that the zygomaticus minor, malaris, and bifid zygomaticus major muscles do not produce any detectable changes in the external morphology of the face, in which case there are no implications for MaqFACS. Additional physiological studies on larger samples may further illuminate the ways in which muscle fibers in the zygomatic region act to produce facial movement.

Finally, the incisivii muscles are linked to the lip pucker movements (AU18i and AU18ii) in MaqFACS. We did not score the incisivii muscles in this study because they were difficult to define and separate from the orbicularis oris and levator anguli oris muscles. Huber (1933) also notes that the incisivii muscles were not distinct in the rhesus macaque. A detailed anatomical study of the muscles in the oral region could provide more details on the possible presence of the incisivii muscles and their role in facial movement.

We found low inter‐individual variation in facial muscle presence, but despite this limited variation, there are individual differences in facial expressivity in this population of rhesus macaques (Whitehouse et al., 2024). Which anatomical structures, if any, produce this behavioral variation remains unclear. It is possible that these individual differences in behavior are the result of inter‐individual variation in muscle morphology (like size or fiber type), neurovascular anatomy, or the superficial musculoaponeurotic system (SMAS) layer (Burrows, Rogers‐Vizena, et al., 2016), rather than muscle presence. Perhaps these individual differences simply result from how the individuals use their muscles (e.g., facial muscle control or the way the behave in social contexts), rather than from differences in musculature. Variation in other aspects of facial morphology, including the distribution of fat and hair, may also influence the production and detection of AUs. Further studies are necessary to uncover the anatomical, physiological, and cognitive basis of variation in facial expressivity in rhesus macaques. For example, comparing variability in both anatomical structures and facial expressivity across species, in relation to social structures, could be useful. If species do not vary in facial anatomy, but do exhibit differences in expressivity, this could result from more flexible and varying social factors in the absence of morphological differences.

Our data show a mix of similarities and differences with data on variation in human facial muscle presence. The upper face exhibits little inter‐individual variation in muscle presence in humans; the frontalis, procerus, and corrugator supercilii muscles are almost always present (Abramo et al., 2016; Costin et al., 2016; Janis et al., 2007), but see Waller, Cray, and Burrows (2008) for exceptions. Similarly, our data show that these muscles are nearly always present in rhesus macaques. In our sample, the most variable region at the gross anatomical level was the midface, specifically the zygomatic region. Humans also exhibit variation in the midface. Multiple configurations of zygomaticus major/minor and the presence of variant muscles, like the malaris muscle (sometimes considered a band of the orbicularis oculi muscle), have been reported at different frequencies in multiple human samples (Choi, Hur, et al., 2014; Farahvash et al., 2010; Park et al., 2011; Pessa et al., 1998). Humans also have a variably present risorius muscle (Choi, Kim, et al., 2014; D'Andrea & Barbaix, 2006), but we did not observe the risorius muscle at all in our rhesus macaque sample. The levator labii superioris alaeque nasi muscle was variably present in some human samples (Sato, 1968; Waller, Cray, & Burrows, 2008), and always present in others (Hur et al., 2010; Kampan et al., 2018). In our sample, this muscle was always present. Although we found two possible occurrences of the malaris muscle and one occurrence of a bifid zygomaticus major insertion, we did not observe most of the variation reported in the human midface. It is possible that the great prognathism in rhesus macaques, compared to humans, influenced the relatively higher level of variation in the zygomaticus muscle compared to other muscles. This could be due to the accelerated growth of the midface during macaque ontogeny (e.g., crab‐eating macaques [*Macaca fasiscularis*] [Nanda et al., 1987], pig‐tailed macaques [*Macaca nemestrina*] [Zumpano & Richtsmeier, 2003]) which may influence the ultimate variable form of the zygomaticus muscle and the surrounding musculature.

In our rhesus macaque sample, there was very little variation in the lower face, consistent with previous data from human cadaveric samples. The mentalis and platysma muscles are always present in human samples (D'Andrea & Barbaix, 2006; Waller, Cray, & Burrows, 2008). The depressor anguli oris and depressor labii inferioris muscles are often, but not always, present in humans (Bae et al., 2016; D'Andrea & Barbaix, 2006; Pessa et al., 1998). Comparative data on inter‐individual variation in facial muscle presence and facial movement in other primate species, especially hominoids, may provide insight into whether the presence of variation in observed facial muscle presence morphology in humans is unique to our species or shared with our closest living relatives. Importantly, it is unclear whether within‐species variation offers an advantage or disadvantage in terms of communication and expressivity. It seems intuitive that uniformity across species is advantageous as it ensures consistent patterns of communication, but it is possible that population‐level variability could be maintained through frequency‐dependent selection.

Our study has some limitations. We measured inter‐individual variation at the gross anatomical level, based on muscle presence. There are likely individual differences in facial muscle size, shape, and physiology that can only be measured at finer scales. We attempted to measure muscle areas from images of the facial masks, but the borders of muscles were challenging to visualize and define, due in part to their attachments to one another, and some muscles, particularly the buccinator, platysma, and frontalis muscles, were always incomplete due to necropsy procedures. Staining methods, including potassium iodide/iodine stain, to facilitate both gross‐level observations (Bock & Shear, 1972) and microCT imaging (Burrows et al., 2019) could help visualize the more gracile muscles and facilitate delineating muscle boundaries. Three‐dimensional surface scanning might also reveal muscle morphology that is difficult to visualize with dissection alone (Weldon et al., 2024). Results from histological studies on muscle fiber type and innervation (Goodmurphy & Ovalle, 1999; Porter et al., 1989) may also help clarify the muscular basis of individual differences in facial expressivity.

The sample size for the anatomy and behavioral correspondence analysis was small, due to the opportunistic nature of our sampling method and the extensive behavioral data required to measure facial behavior using FACS. The small sample size limited our ability to conduct statistical analyses on our data. The sample is also derived from a lab, and may exhibit lower genetic and phenotypic variation than a wild population. Due to the demographics of the lab population, our sample also included far more females than males. Future studies on larger samples could explore age and sex effects, including the impact of sexual dimorphism on facial musculature and facial behavior. Finally, we integrated antemortem data on behavior with postmortem dissection data, which limits our ability to make direct correlations between facial musculature and facial behavior during life. An in vivo imaging study using ultrasound or magnetic resonance imaging (see Franchi et al., 2018) of human facial behavior could validate relationships between muscle action and external movements of the face.

Using the largest sample to date of non‐human primate faces from a single species, we show that rhesus macaques exhibit limited inter‐individual variation in facial muscle presence. Our data suggest that rhesus macaques exhibit less overall inter‐individual variation in facial muscle presence than humans do. From an evolutionary perspective, this is interesting, as it suggests that the variation present in the human face has evolved relatively recently. Whether this (potentially human unique) variation has an adaptive function is unknown, but worthy of future investigation. We also show that individuals who produced MaqFACS AU possessed the proposed muscular basis for that AU, which supports the use of the FACS approach to measuring facial behavior. Our data suggest that observed individual differences in rhesus macaque facial behavior cannot be explained by individual differences in muscle presence alone. Anatomical, physiological, and behavioral studies of facial expressivity in other nonhuman primate species will help explain the evolution of complex facial behavior in humans.

## AUTHOR CONTRIBUTIONS

**Clare M. Kimock:** Conceptualization; data curation; formal analysis; investigation; methodology; project administration; visualization; writing – original draft. **Charles Ritchie:** Data curation; investigation; methodology; writing – review and editing. **Jamie Whitehouse:** Data curation; investigation; methodology; visualization; writing – review and editing. **Claire Witham:** Resources; writing – review and editing. **Claire M. Tierney:** Conceptualization; funding acquisition; resources; supervision; writing – review and editing. **Nathan Jeffery:** Resources; supervision; writing – review and editing. **Bridget M. Waller:** Conceptualization; funding acquisition; investigation; methodology; supervision; writing – review and editing. **Anne M. Burrows:** Conceptualization; investigation; methodology; supervision; writing – review and editing.

## Supporting information


**Data S1:** Supporting Information.

## Data Availability

The data that support the findings of this study are openly available on the Open Science Framework at https://osf.io/4ufpk/?view_only=c9becb2b182c43269f51c7830eb15426.
