# Supplementary material for: Linking individual variation in facial musculature to facial behavior in rhesus macaques
Source: Anat Rec (Hoboken). 2025 Mar 17;308(12):3105–21. doi: 10.1002/ar.25650 (PMC12594182; doi:10.1002/ar.25650)
Supplement: Supplementary file 1 — Data S1: Supporting Information. [file AR-308-3105-s001.docx]

**Supplemental Information for “Linking individual variation in facial musculature to facial behaviour in rhesus macaques”**

Minimal observation time needed for reliable AU detection

To calculate how much observation time was needed to reliably detect AUs (in order to use as our minimal observation time threshold), we generated smaller subsets of data in increasing time intervals and assessed those data for the presence of each AU. We generated 34 subsets of data, increasing in 300 second (5 minute) intervals from 0-10000 seconds; repeating the process over 500 iterations. We calculated a detection rate for each time interval, which was the proportion of samples containing each AU across the 500 iterations (e.g. a detection rate of 0.5 would suggest the AU was present in 250 of the randomly generated samples for that time interval).

At 3600 seconds (1 hour) of footage, 17 of 19 AUs (with the exception of AU17 and AU8) had a detection rate of 70% or higher, with most having a detection rate of 95%+ (Supplemental Figure 1). As a result, we decided this was an appropriately cautious cut-off threshold for minimum observation time.

| 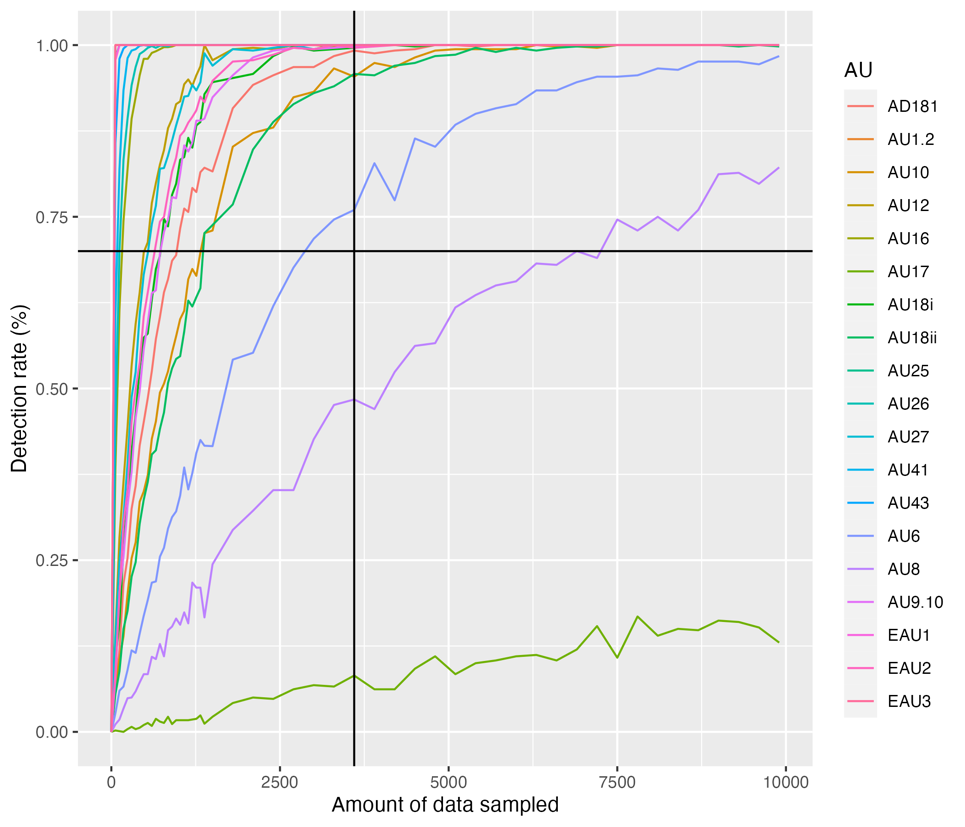 |
| --- |
| **Supplemental Figure 1.** The detection rate of each AU across observation time. The y axis corresponds to observation time of the sampled data, and the y axis corresponds to how often each AU was detected across 500 randomly generated samples. The horizonal solid line represents a detection rate threshold of 70%. The vertical solid line represents 3600 seconds (1 hour) of observation time. |

Asymmetry in action unit expression


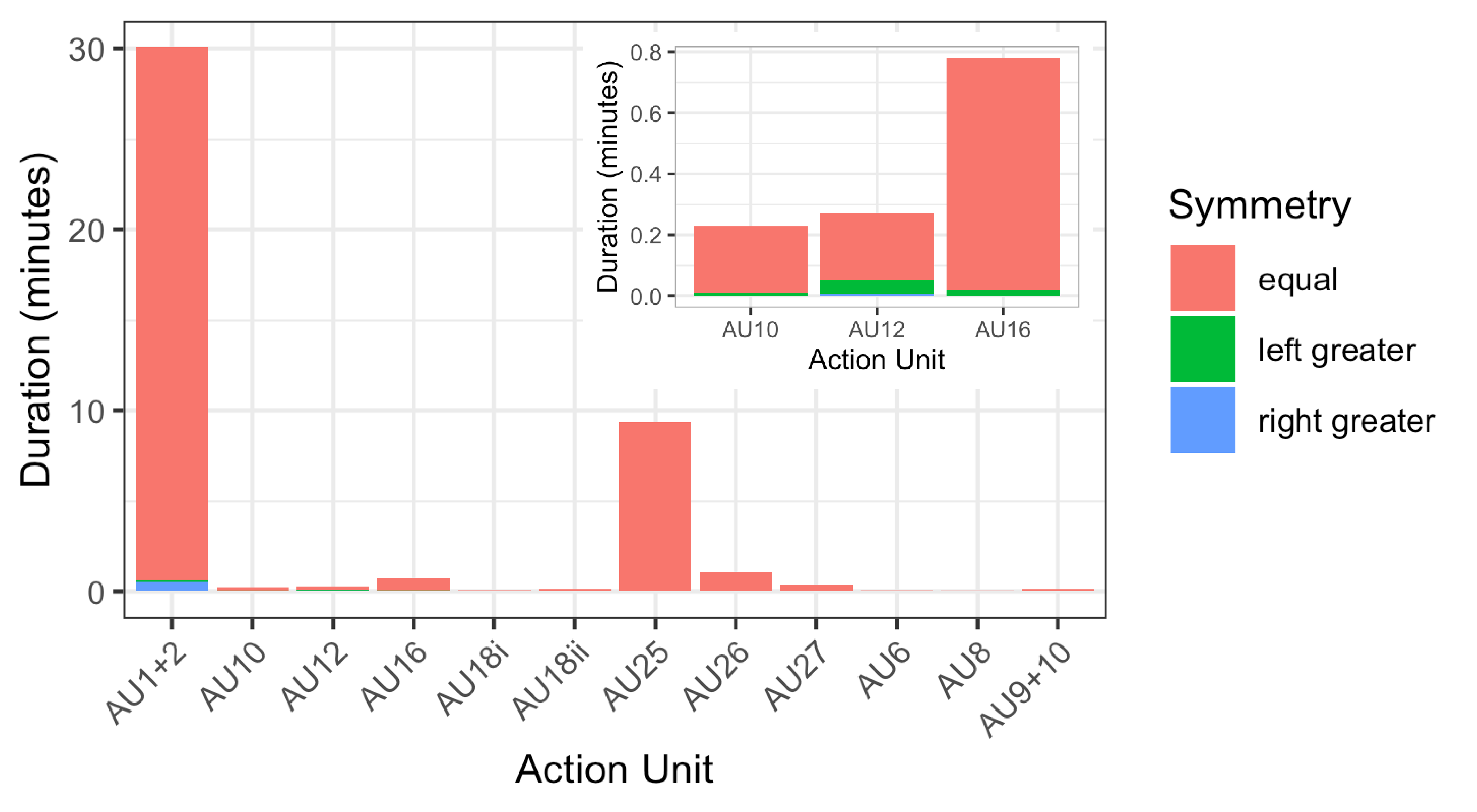

**Supplemental Figure 2**. Plot of asymmetry in AU duration from a subset of the video data. Data are from the eight individuals included in the behaviour and anatomy correspondence analysis.
